# Supplementary material for: Exploring the Potentiality of a Plant Platform for Monoclonal Antibody Production in Veterinary Medicine
Source: Vaccines (Basel). 2024 Jun 4;12(6):620. doi: 10.3390/vaccines12060620 (PMC11209382; doi:10.3390/vaccines12060620)
Supplement: Supplementary file 1 [file vaccines-12-00620-s001.zip › vaccines-3014870-supplementary.pdf]

SUPPLEMENTARY INFORMATION

Exploring the potentiality of a plant platform for monoclonal antibody production in veterinary medicine

B. Morel [1], C. Favrot [2, *], L. Mirande [1], C. Grünwald-Gruber [3], V. Stordeur [1], L. Vezina [4], L. Faye [1], V. Gomord [1,4,*]

[1] ANGANY Innovation, 1 voie de l'innovation, 27100 Val de Reuil, France

[2] Dermatology Unit, Clinical for Small Animal Internal Medicine, Vetsuisse Faculty, University of Zurich, Switzerland

[3] BOKU Core Facility Mass Spectrometry, BOKU University, Muthgasse 18, A-1190 Vienna, Austria

[4] ANGANY Inc, Suite 200, 873 St-Jean, Québec, QC G1R 1R2, Canada

* Corresponding authors : VG : Veronique.gomord@angany.com; CF : favrot68@gmail.com

Type of paper: Article

Table S1. Relative proportions of detected glycoforms.

	M1 WT	M1 ΔXF	Lokivetmab
Notglyc	4.93%	3.53%	0.79%
Man5	7.33%	6.45%	0.65%
Man6	4.59%	4.33%	0.08%
Man7	11.69%	11.45%	0.04%
Man8	11.76%	13.29%	0.10%
Man9	8.74%	8.46%	0.01%
Man9+Hex	0.43%	0.34%	0.00%
Man5Gn	0.55%	8.01%	0.02%
Man5Gn+1xHex	0.18%	0.84%	0.00%
GnUF	0.13%		2.22%
MMF	0.36%		0.12%
MGnF	0.89%		5.74%
GnGnF	8.93%		78.87%
AMF	0.51%		0.15%
AGnF	4.41%		3.82%
AAF	1.79%		0.12%
MM	0.12%	0.19%	0.06%
GnM	0.85%	5.28%	1.22%
GnGn	12.58%	30.51%	5.42%
AGn	0.46%	2.69%	0.52%
AA	0.19%	0.29%	0.02%
MMXF	0.63%		
GnMXF	1.68%		
GNGNXF	15.86%		
Man4Gn/AM	0.40%	4.35%	0.04%

(a)

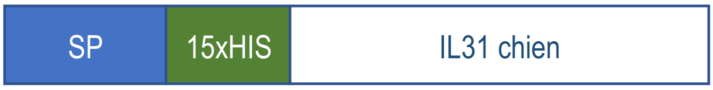

(b)

SP-Variable light chain lokivetmab - constant λ

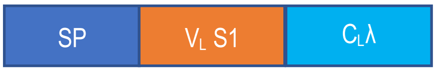

SP-variable heavy - const Heavy

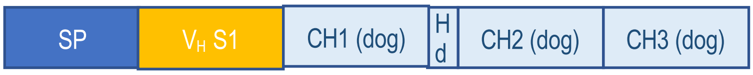

Figure S1. Schematic representation of the constructs used in this study. cIL-31 (Plant) (a) and M1 (b). SP stands for signal peptide

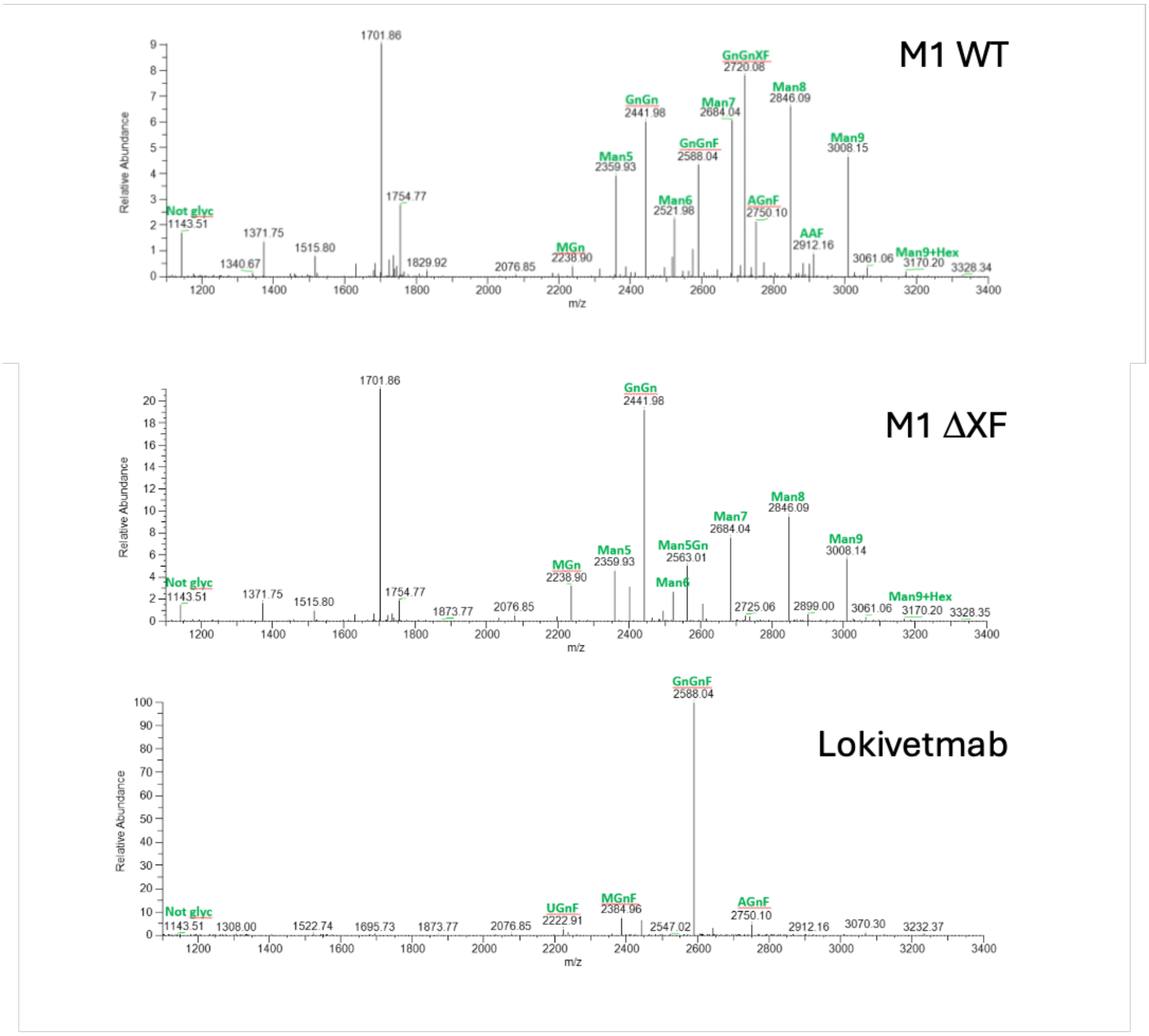

Figure S2. Liquid chromatography mass spectrometry (LC/MS) analysis illustrating the N-glycosylation profiles of the heavy chain peptide EEQFNGTYR (mass: 1143.51 Da) from plant produced M1 antibodies (WT, ΔXF) and lokivetmab produced in CHO.

Raw data of the Western Blot presented in the main text body.

Figure 1.

Date:	2022/01/27 12:30:11 PM
Mode:	Chemi Blots
Notes:	No Comments
Model:	iBright™ FL1500
Inst Name:	2462620120020
Serial No:	2462620120020
Version:	1.6.0
User:	Bertrand
Exposure Time:	10000 ms
Exposure Mode:	Normal
Image area:	112.70mm x 90.16mm
Image size	676px x 540px
Optical Zoom:	2x
Digital Zoom:	1x
Focus level:	450
Dye:	Chemi
Excitation:	None
Emission:	None
Resolution:	5 x 5
Sensitivity:	100%

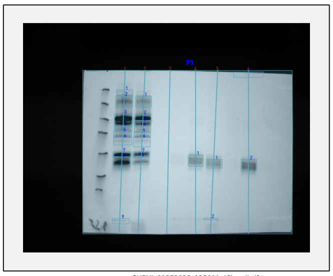

CHEMI_01272022_123011_(Chemi).tif

CHEMI_01272022_123011_(Chemi).tif
LANE AND BAND ANALYSIS DATA TABLE

Frame 1 Channel 1
Lane 1

Band ID	Volume	Area	Local Bg. Corr. Vol.	Rf	Density	Band Purity %
1	9290710	600	1954460	0.135	15484	1.909
2	30052000	1092	14851400	0.184	27520	14.502
3	65654900	1320	42728400	0.298	49739	41.724
4	15800000	602	1795900	0.352	26246	1.754
5	15154900	660	4714130	0.389	22962	4.603
6	12583100	855	895047	0.430	14717	0.874
7	27348200	572	15432700	0.510	47812	15.070
8	47067000	1050	18562700	0.557	44826	18.127
9	3630170	574	1471620	0.917	6324	1.437

Lane 2

Band ID	Volume	Area	Local Bg. Corr. Vol.	Rf	Density	Band Purity %
1	23635200	1014	11367900	0.187	23309	19.981
2	33125000	697	17186100	0.285	47525	30.207
3	21597400	533	6612800	0.321	40520	11.623
4	14033300	640	1489590	0.355	21927	2.618
5	11129300	520	3855940	0.391	21402	6.777
6	12950500	984	1237240	0.435	13161	2.175
7	18486300	480	11191900	0.508	38513	19.672
8	19891500	840	3952330	0.549	23680	6.947

Lane 4

Band ID	Volume	Area	Local Bg. Corr. Vol.	Rf	Density	Band Purity %
1	22849100	1073	12985000	0.549	21295	100

Lane 5

Band ID	Volume	Area	Local Bg. Corr. Vol.	Rf	Density	Band Purity %
1	16515400	858	8190650	0.567	19249	94.185
2	1840000	216	505665	0.904	8518	5.815

Lane 6

Band ID	Volume	Area	Local Bg. Corr. Vol.	Rf	Density	Band Purity %
1	5554480	1008	2497560	0.023	5510	11.623
2	29785800	1170	18990800	0.578	25458	88.377

Figure 2.

Date: 2023/08/16 02:37:23 PM
Mode: Chemi Blots
Notes: No Comments
Model: iBright™ FL1500
Inst Name: 2462620120020
Serial No: 2462620120020
Version: 1.8.0
User: M1_Modifie
Exposure Time: 613 ms
Exposure Mode: Normal
Image area: 118.63mm x 94.91mm
Image size 676px x 540px
Optical Zoom: 1.9x
Digital Zoom: 1x
Focus level: 430
Dye: Chemi
Excitation: None
Emission: None
Resolution: 5 x 5
Sensitivity: 100%

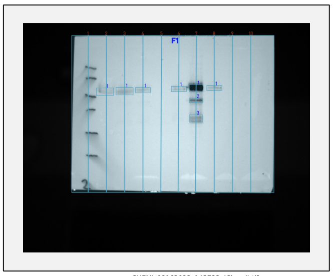

CHEMI_08162023_143723_(Chemi).tif

CHEMI_08162023_143723_(Chemi).tif
LANE AND BAND ANALYSIS DATA TABLE

Frame 1 Channel 1
Lane 2

Band ID	Volume	Area	Local Bg. Corr. Vol.	Rf	Density	Band Purity %
1	1396870	798	991483	0.356	1750	100

Lane 3

Band ID	Volume	Area	Local Bg. Corr. Vol.	Rf	Density	Band Purity %
1	2209440	798	1712310	0.353	2768	100

Lane 4

Band ID	Volume	Area	Local Bg. Corr. Vol.	Rf	Density	Band Purity %
1	1184410	555	946748	0.348	2134	100

Lane 6

Band ID	Volume	Area	Local Bg. Corr. Vol.	Rf	Density	Band Purity %
1	1471140	555	792614	0.340	2650	100

Lane 7

Band ID	Volume	Area	Local Bg. Corr. Vol.	Rf	Density	Band Purity %
1	16426400	700	13634300	0.334	23466	72.858
2	3506970	442	2433190	0.412	7934	13.002
3	3794800	680	2645970	0.526	5580	14.139

Lane 8

Band ID	Volume	Area	Local Bg. Corr. Vol.	Rf	Density	Band Purity %
1	1172830	532	922622	0.332	2204	100

Figure 3.

Date: 2022/12/02 02:19:18 PM
Mode: Chemi Blots
Notes: No Comments
Model: iBright™ FL1500
Inst Name: 2462620120020
Serial No: 2462620120020
Version: 1.8.0
User: M1CYT
Exposure Time: 26210 ms
Exposure Mode: Normal
Image area: 132.59mm x 106.07mm
Image size 676px x 540px
Optical Zoom: 1.7x
Digital Zoom: 1x
Focus level: 380
Dye: Chemi
Excitation: None
Emission: None
Resolution: 5 x 5
Sensitivity: 100%

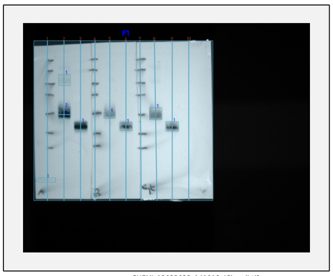

CHEMI_12022022_141918_(Chemi).tif

CHEMI_12022022_141918_(Chemi).tif
LANE AND BAND ANALYSIS DATA TABLE

Frame 1 Channel 1
Lane 1

Band ID	Volume	Area	Local Bg. Corr. Vol.	Rf	Density	Band Purity %
1	616120	576	415416	0.865	1069	100

Lane 2

Band ID	Volume	Area	Local Bg. Corr. Vol.	Rf	Density	Band Purity %
1	2690250	840	2100610	0.241	3202	13.014
2	15717900	589	8178460	0.434	26686	50.669
3	10116700	341	5861920	0.471	29668	36.317

Lane 3

Band ID	Volume	Area	Local Bg. Corr. Vol.	Rf	Density	Band Purity %
1	20076700	660	16976000	0.529	30419	100

Lane 5

Band ID	Volume	Area	Local Bg. Corr. Vol.	Rf	Density	Band Purity %
1	5480280	464	3597060	0.463	11811	100

Lane 6

Band ID	Volume	Area	Local Bg. Corr. Vol.	Rf	Density	Band Purity %
1	10404300	672	8877920	0.534	15482	100

Lane 8

Band ID	Volume	Area	Local Bg. Corr. Vol.	Rf	Density	Band Purity %
1	11872200	960	10154800	0.452	12367	100

Lane 9

Band ID	Volume	Area	Local Bg. Corr. Vol.	Rf	Density	Band Purity %
1	11162300	768	10216800	0.532	14534	100
